# Supplementary figures and images for: Hookworms dynamically respond to loss of Type 2 immune pressure
Source: PLoS Pathog. 2023 Dec 11;19(12):e1011797. doi: 10.1371/journal.ppat.1011797 (PMC10735188; doi:10.1371/journal.ppat.1011797)

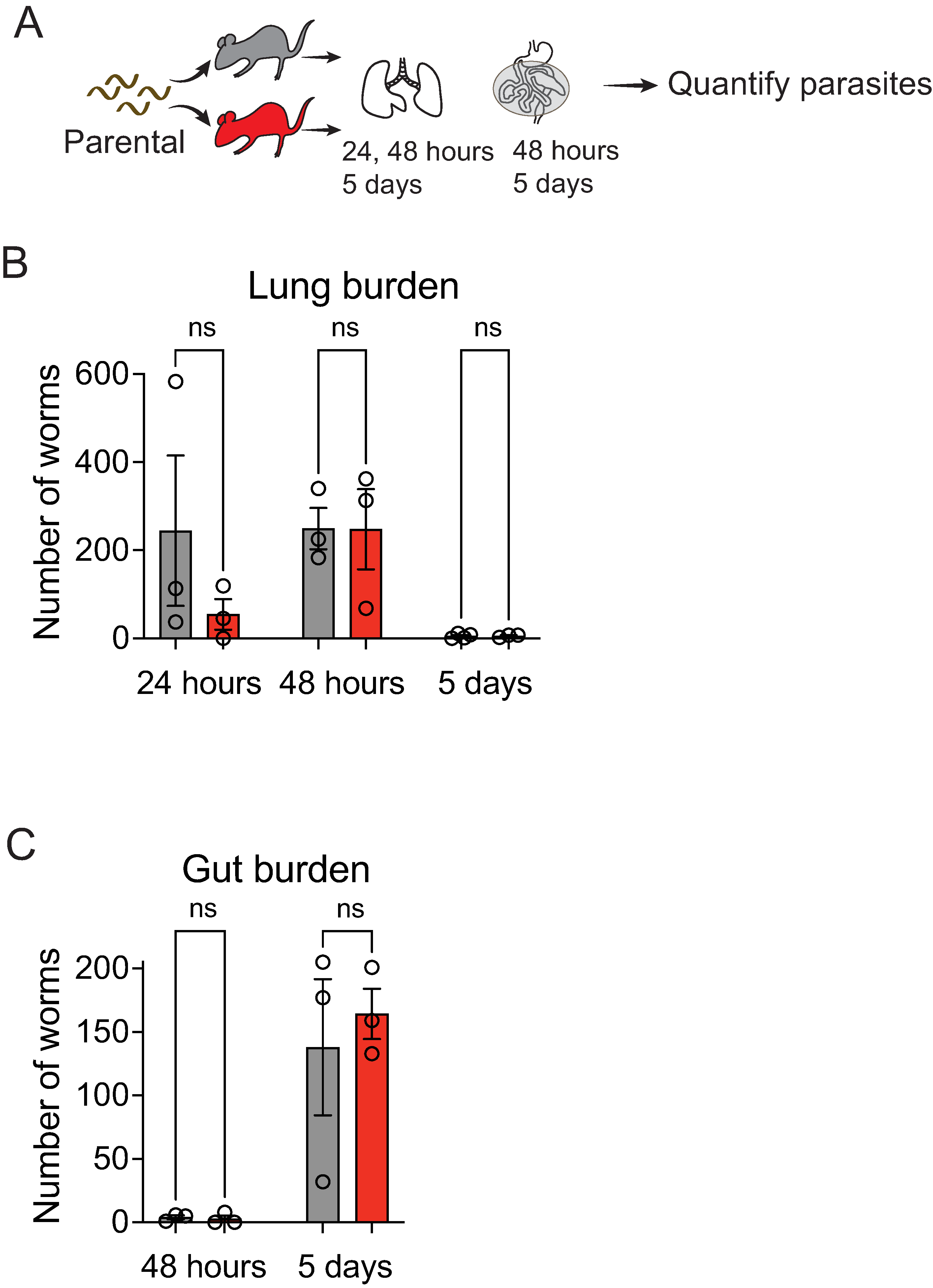

Supplement: S1 Fig — A. Experimental design. B. Number of parasites in the lungs after 24 hours, 48 hours, or 5 days post infection. C. Number of parasites in the gut after 48 hours or 5 days post infection. Each point is a replicate mouse, and error bars are SEM. ns p-value > 0.05, by t-test. Data are representative of 1 independent experiment. (TIF) [file ppat.1011797.s001.tif]

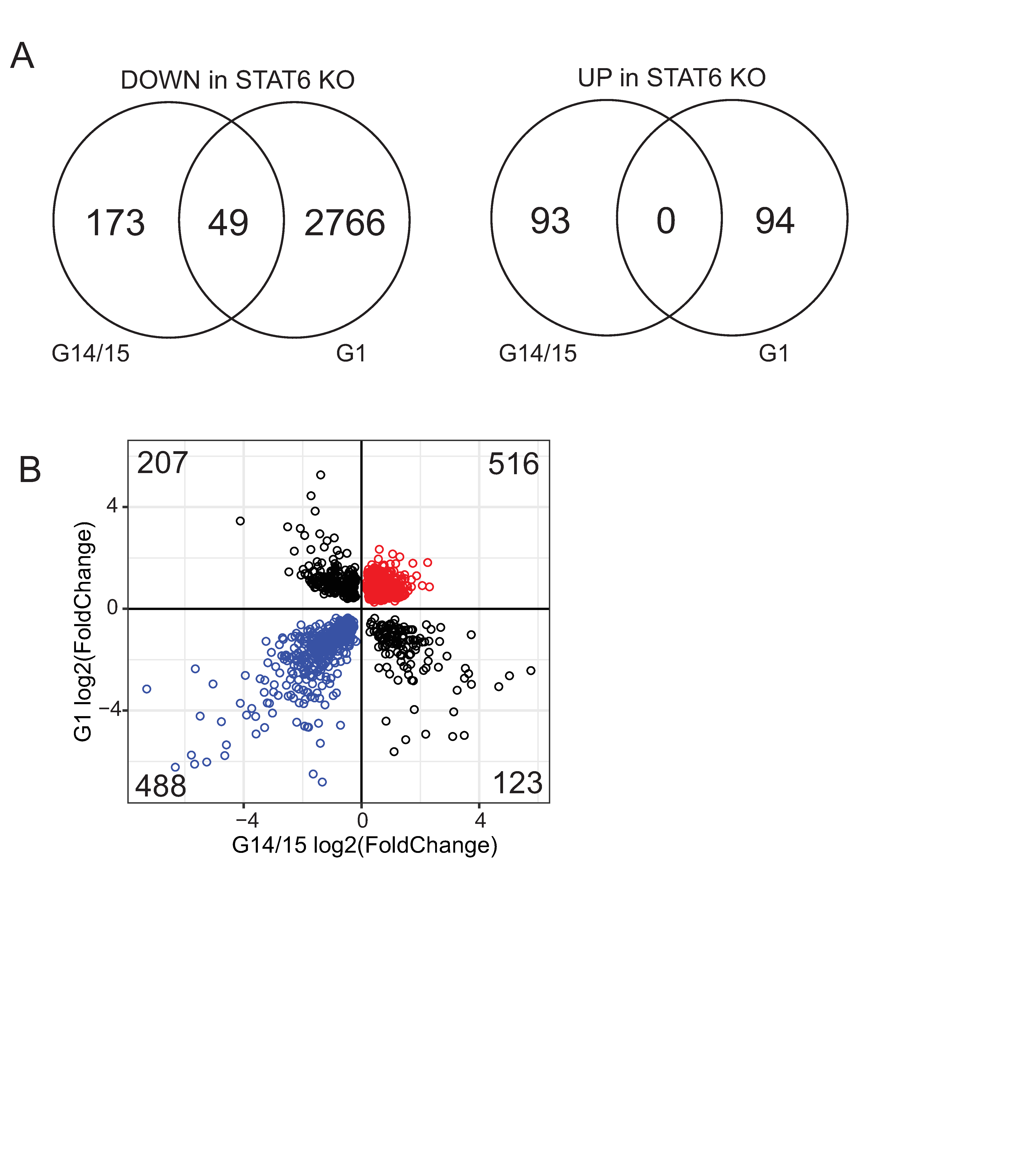

Supplement: S2 Fig — A. Venn diagrams of significantly up (Log2FoldChange > 2) versus down (Log2FoldChange < -2) regulated genes for STAT6 KO versus WT host conditions (for both, adjusted p < 0.05). B. Scatterplot of all STAT6 KO vs. WT host condition Log2(FoldChange) values, filtered on adjusted p-value < 0.05, in single generation N. brasiliensis infection, (G1) (y-axis) versus adapted N. brasiliensis (G15/14) (x-axis). Values represent the number of genes in each quadrant. (TIF) [file ppat.1011797.s002.tif]

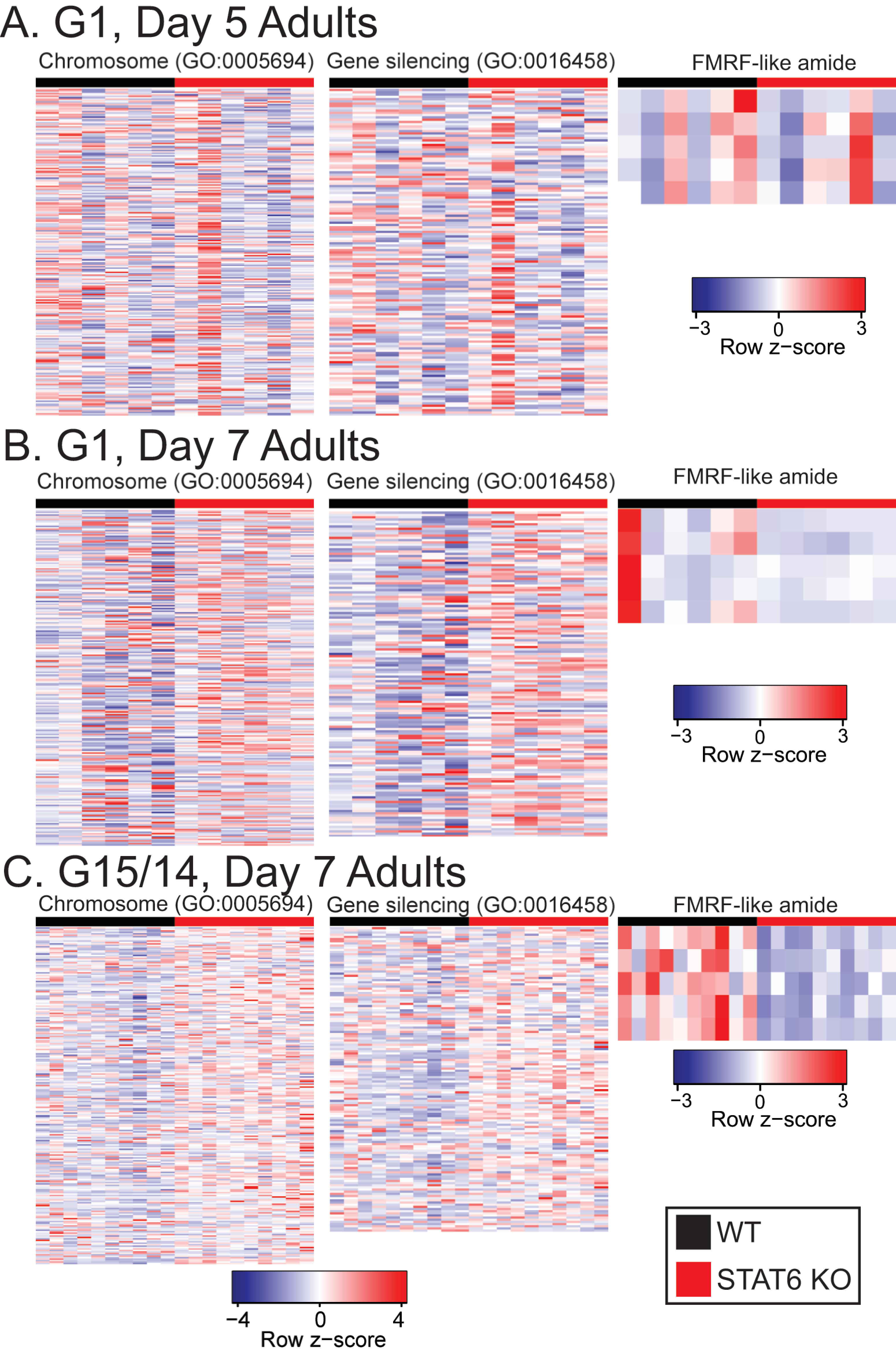

Supplement: S3 Fig — Heatmaps of gene sets of interest from A. Day 5 females WT versus STAT6 KO hosts infected with progeny of parental N. brasiliensis (G1), B. Day 7 females of G1, or C. Day 7 females worms from WT or STAT6 KO hosts infected with G15 or 14 adapted N. brasiliensis respectively. (TIF) [file ppat.1011797.s003.tif]

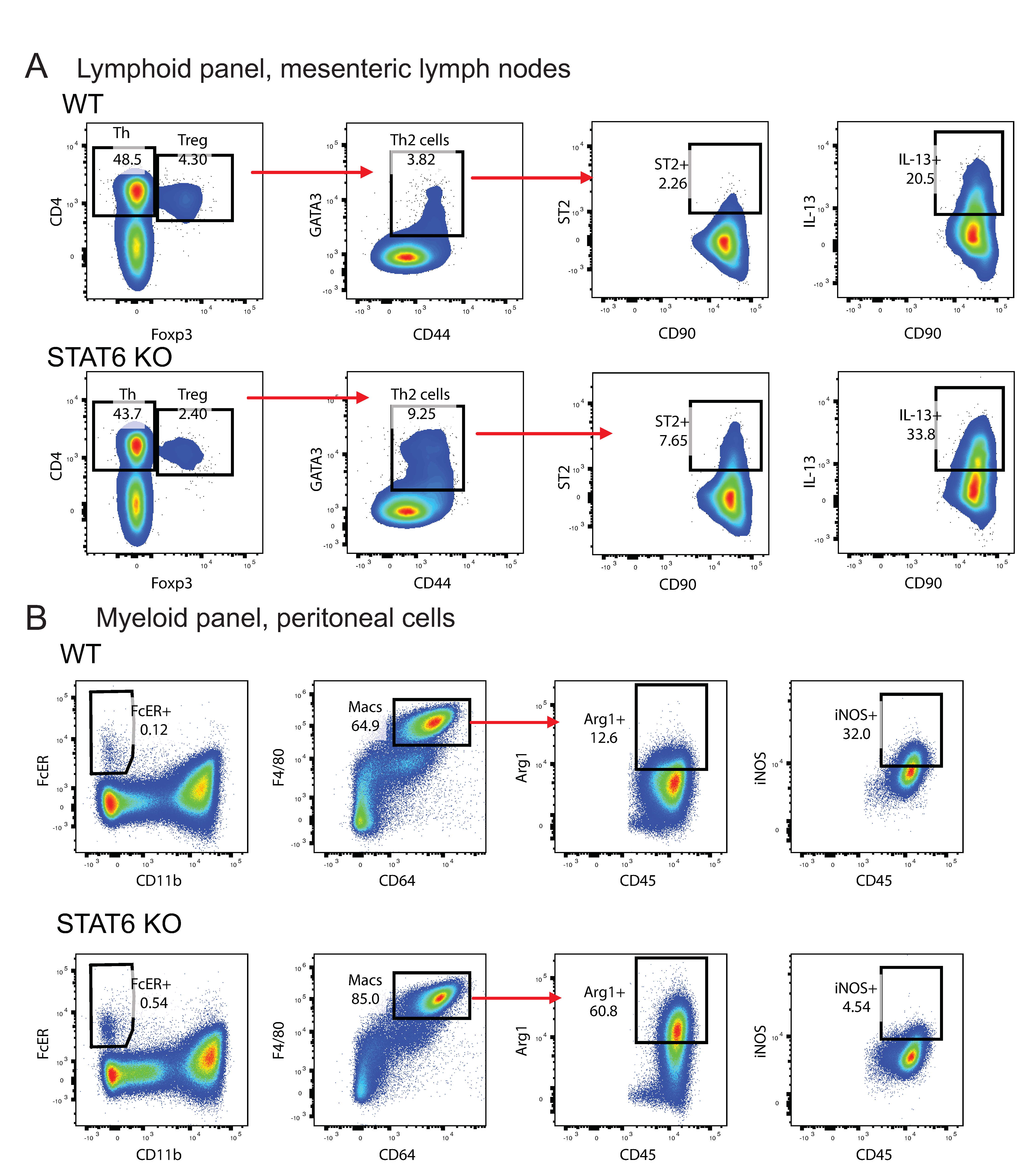

Supplement: S4 Fig — A. Gating strategy to detect lymphoid cell populations from peritoneal cells. B. Gating strategy for detection of myeloid cell populations from mesenteric lymph nodes. (TIF) [file ppat.1011797.s004.tif]

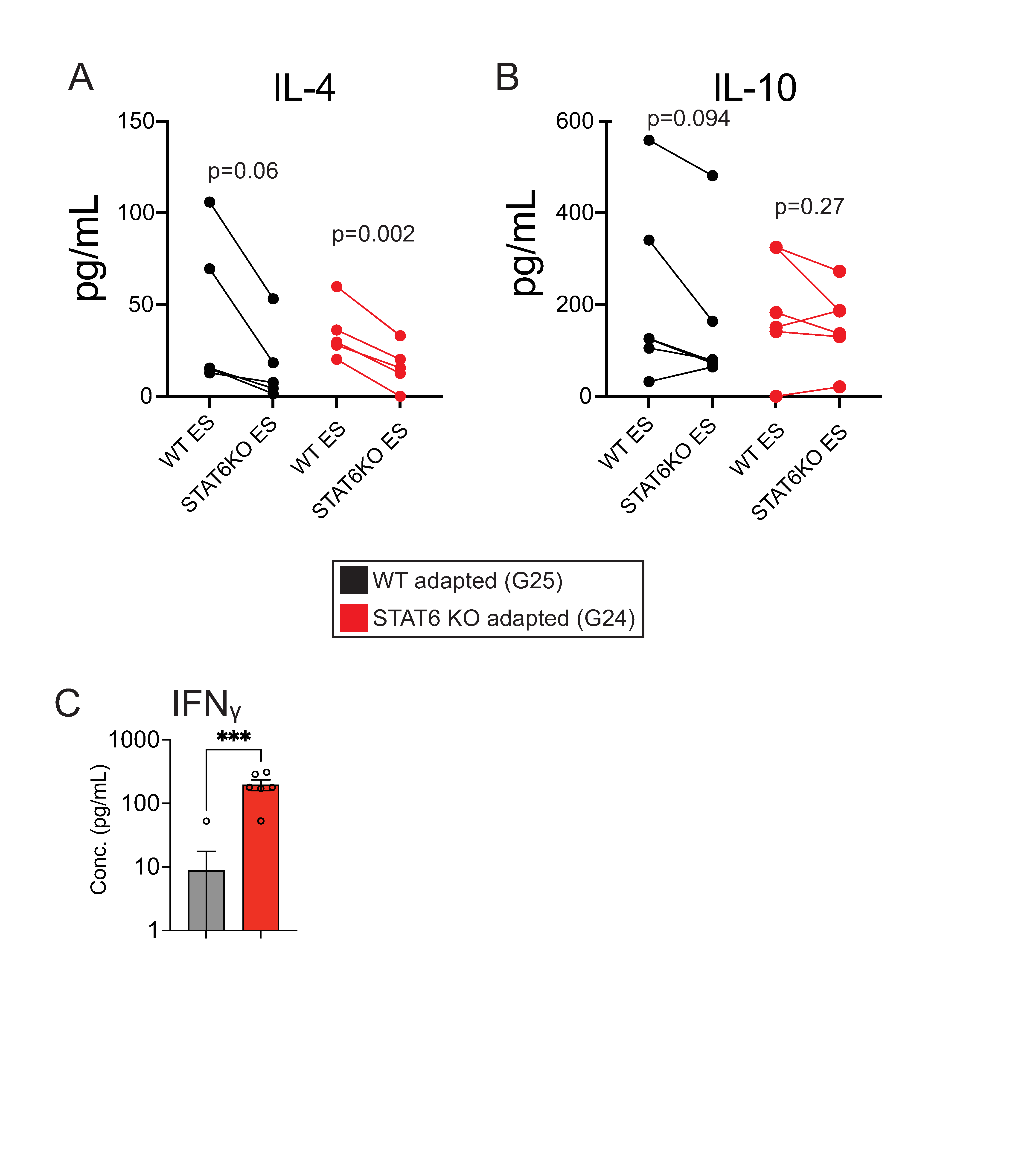

Supplement: S5 Fig — Supernatant from 3-day cultures of WT or STAT6 KO- adapted (G24/23) N. brasiliensis was used to stimulate mesenteric lymph node cells collected from WT mice infected with either WT or STAT6 KO -adapted (G25/24) N. brasilensis at day 8 post infection. A. concentration of IL-4 in supernatant. B. concentration of IL-10 in supernatant. P-values are from paired t-tests. C. Concentration of IFNγ measured following anti-CD3/28 stimulation for each infected host (circles), with SEM error bars. ***p-value < 0.001 by t-test. Data is representative of 2 independent experiments. (TIF) [file ppat.1011797.s005.tif]

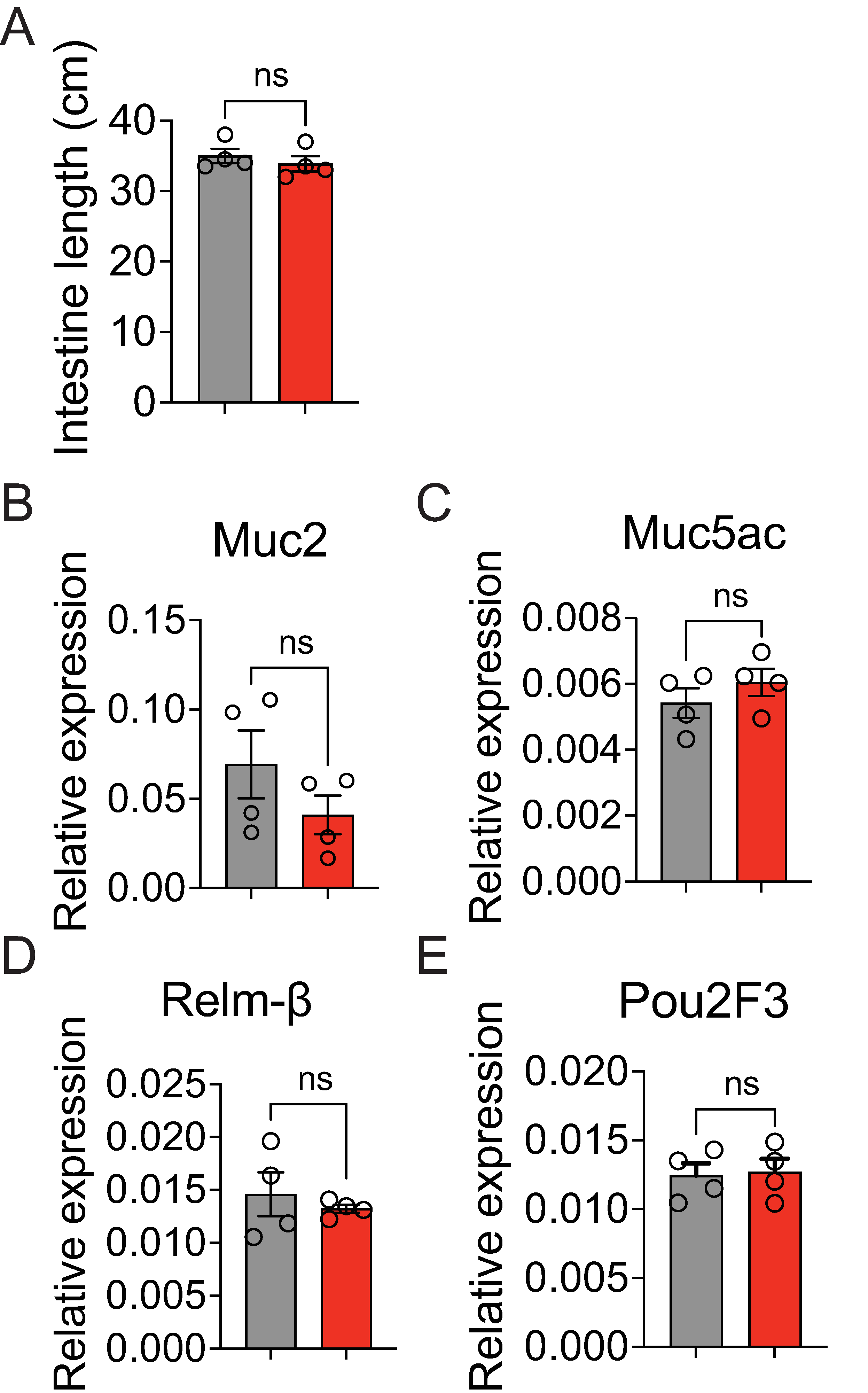

Supplement: S6 Fig — A. Length of intestine. B-E. RT-qPCR expression relative to GAPDH. Circles are individual infected hosts, bars are mean values, ns p-value > 0.05 by t-test. Data is representative of 1 independent experiment. (TIF) [file ppat.1011797.s006.tif]

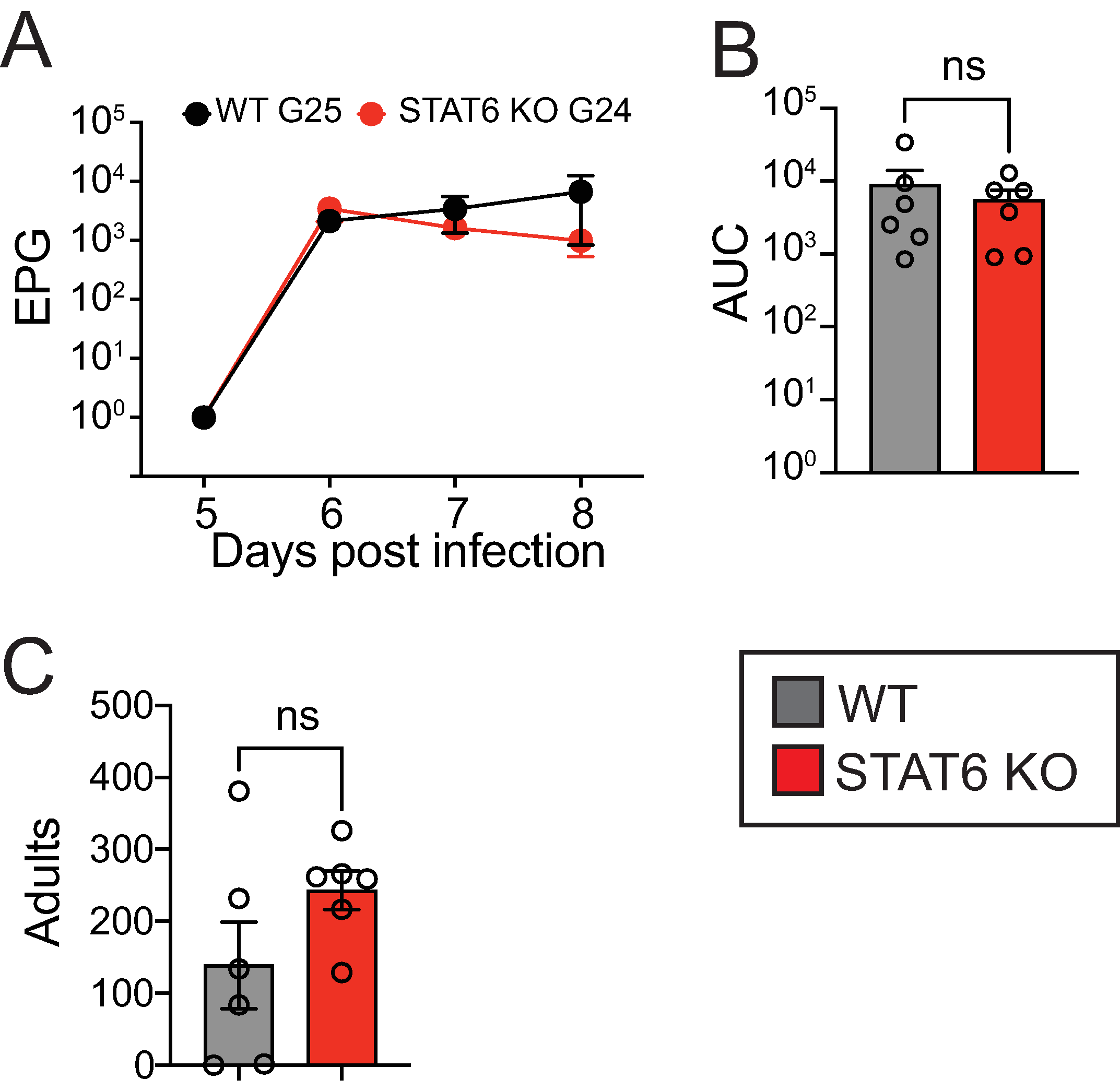

Supplement: S7 Fig — A. Average eggs per gram of feces by days post infection, with SEM error bars. B. Average area under the curve of data plotted in A. C. Number of adult parasites in the small intestine at day 8 post infection. (TIF) [file ppat.1011797.s007.tif]

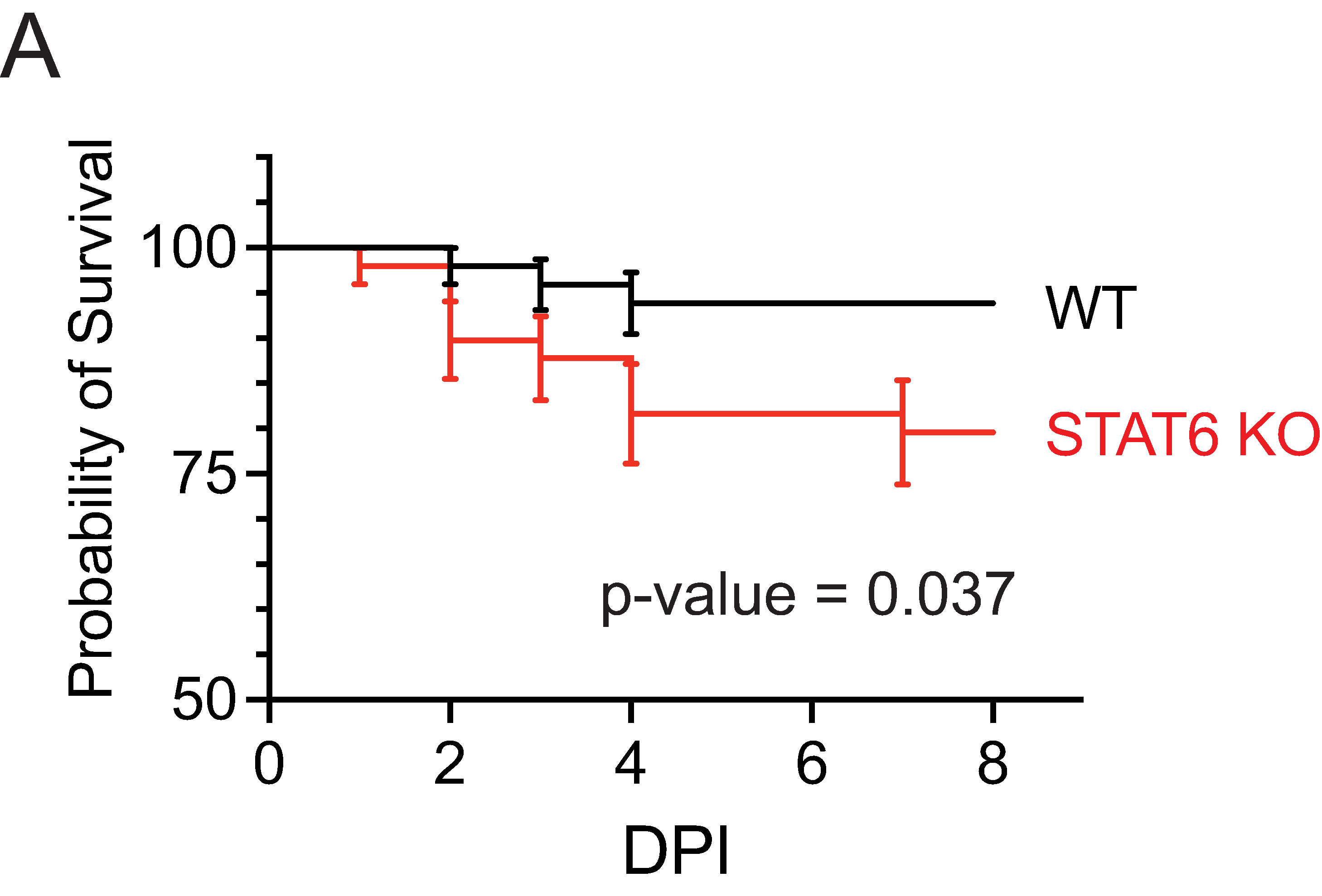

Supplement: S8 Fig — A. Survival curve with average host survival by days post infection for WT hosts infected with WT-adapted or STAT6 KO-adapted worms. Combined data from 9 independent experiments, 49 mice per condition, Mantel-Cox test p-value. (TIF) [file ppat.1011797.s008.tif]

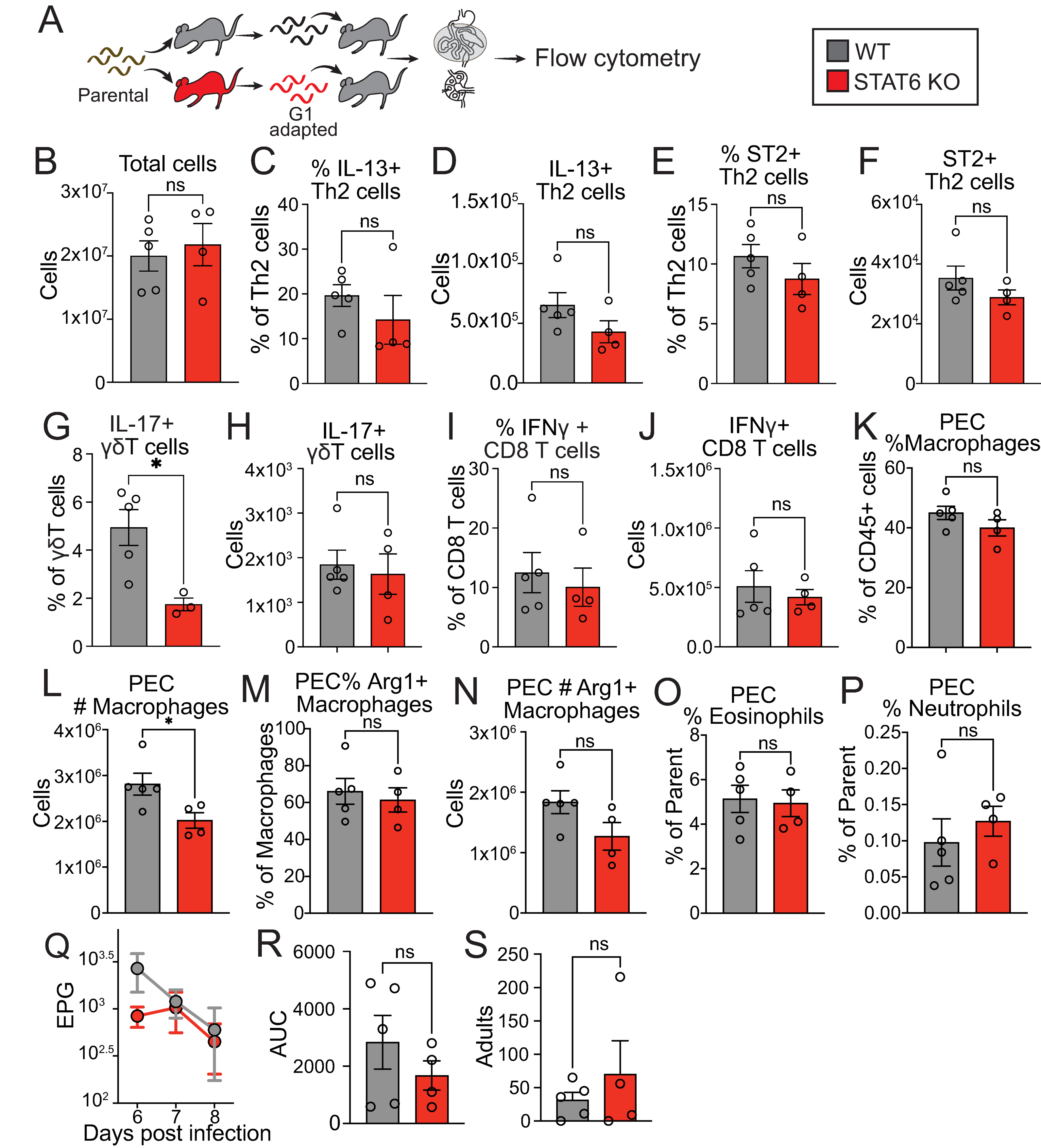

Supplement: S9 Fig — Mesenteric lymph node data (A-J) A. Experimental design; Parental worms infected WT or STAT6 KO mice, and resulting progeny (G1 adapted) infected WT mice. Flow cytometry was run on cells from mesenteric lymph nodes at day 8 post infection. B. Total number of mesenteric lymph node cells. C-D. Percent and total cell number of IL-13+ Th2 cells. E-F. Percent and total cell number of ST2+ Th2 cells. G-H. Percent and total cell number of IL-17+ γδT cells. I-J. Percent and total cell number of IFNγ+ CD8+ T cells in mesenteric lymph nodes. Data from the peritoneal cavity (K-P). K-L. Percentage of parent population or total cell number of macrophages. M-N. Percentage or total cell number of Arg-1 expressing macrophages. O-P. Percentage of parent population of eosinophils and neutrophils. Q. Parasite burden by number of eggs per gram of feces (EPG) for each day post infection. R. Area under the curve. S. Number of worms from host small intestine at day 8 post infection. Each point is a replicate mouse, and error bars are SEM. * p < 0.05 ** p < 0.01, *** p < 0.001, by t-test. Data are representative of 1 independent experiment. (TIF) [file ppat.1011797.s009.tif]

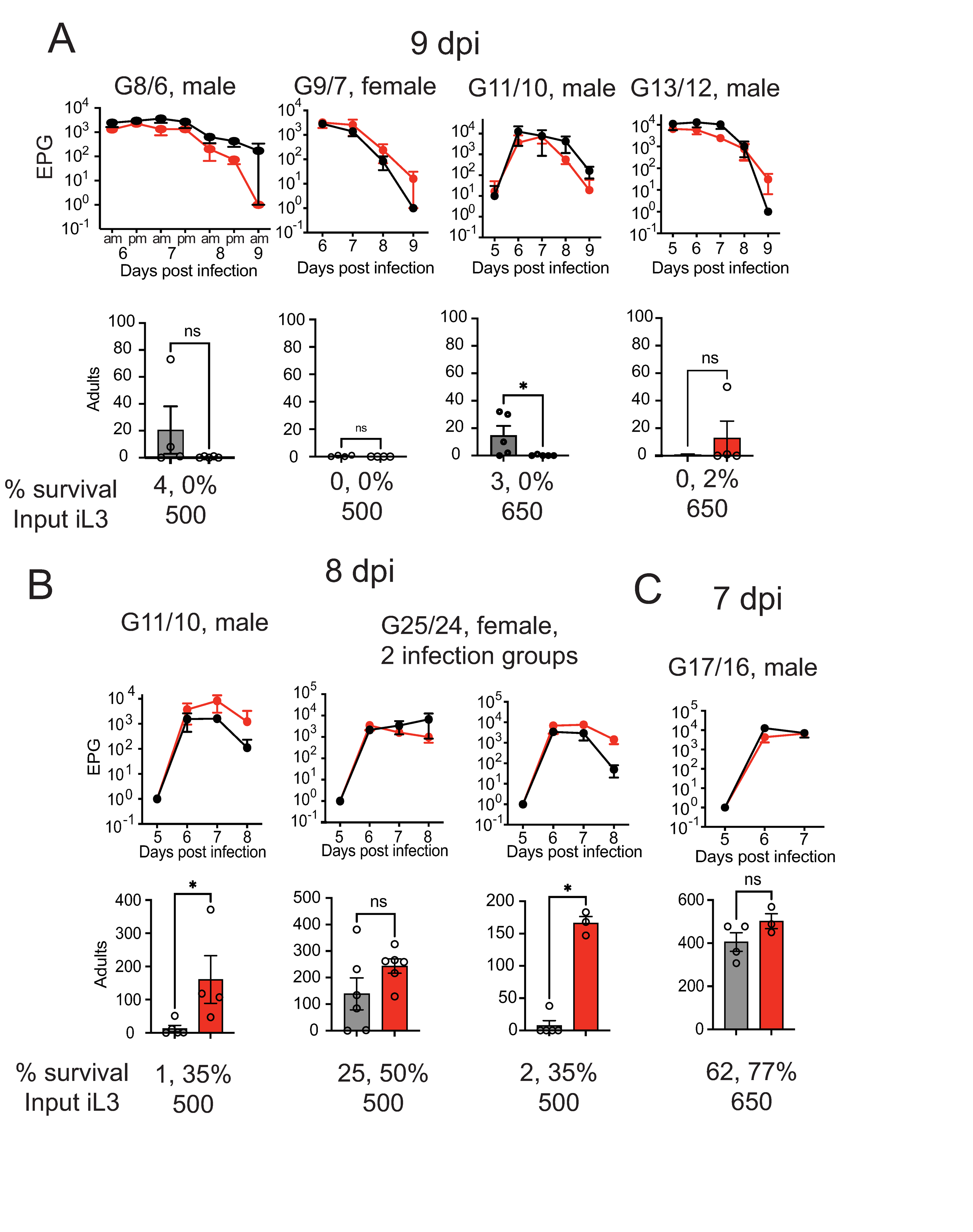

Supplement: S10 Fig — A-C. Plots of eggs per gram of feces (EPG) by days post infection of WT mice infected with STAT6 KO- or WT-adapted N. brasiliensis percentage of surviving adults from the initial inoculum number shown below. Number of generations of adaptation and sex of mice noted as generations in STAT6 KO/WT mice above plots. Data collected until day 9 (A), day 8 (B), or day 7 (C) post infection. (TIF) [file ppat.1011797.s010.tif]

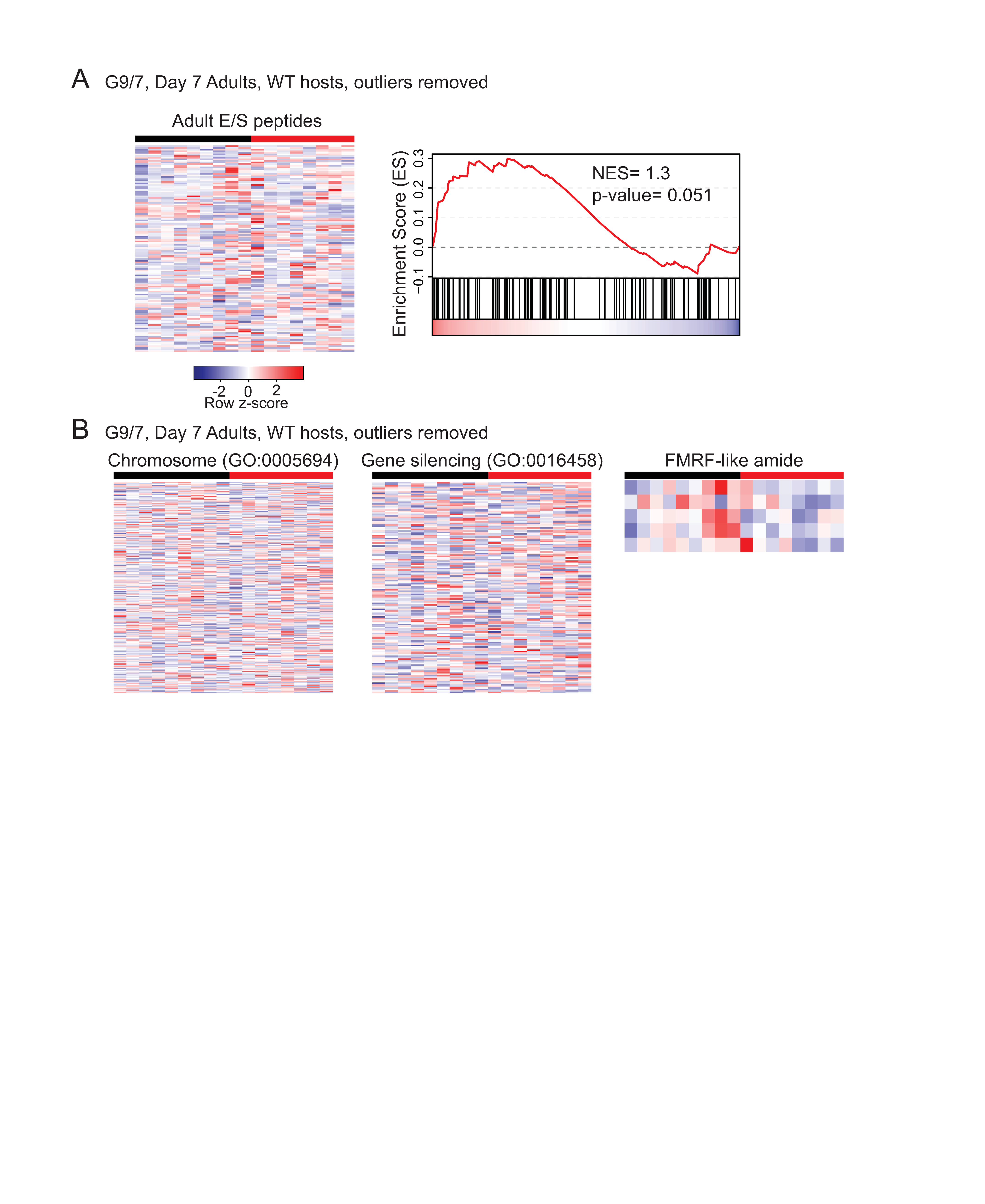

Supplement: S11 Fig — A. Heatmap and GSEA enrichment plot for genes encoding adult stage ESP-associated gene categories. B. Heatmaps for expression of genes in Chromosome, Gene Silencing, or FMRFamide-like families. (TIF) [file ppat.1011797.s011.tif]

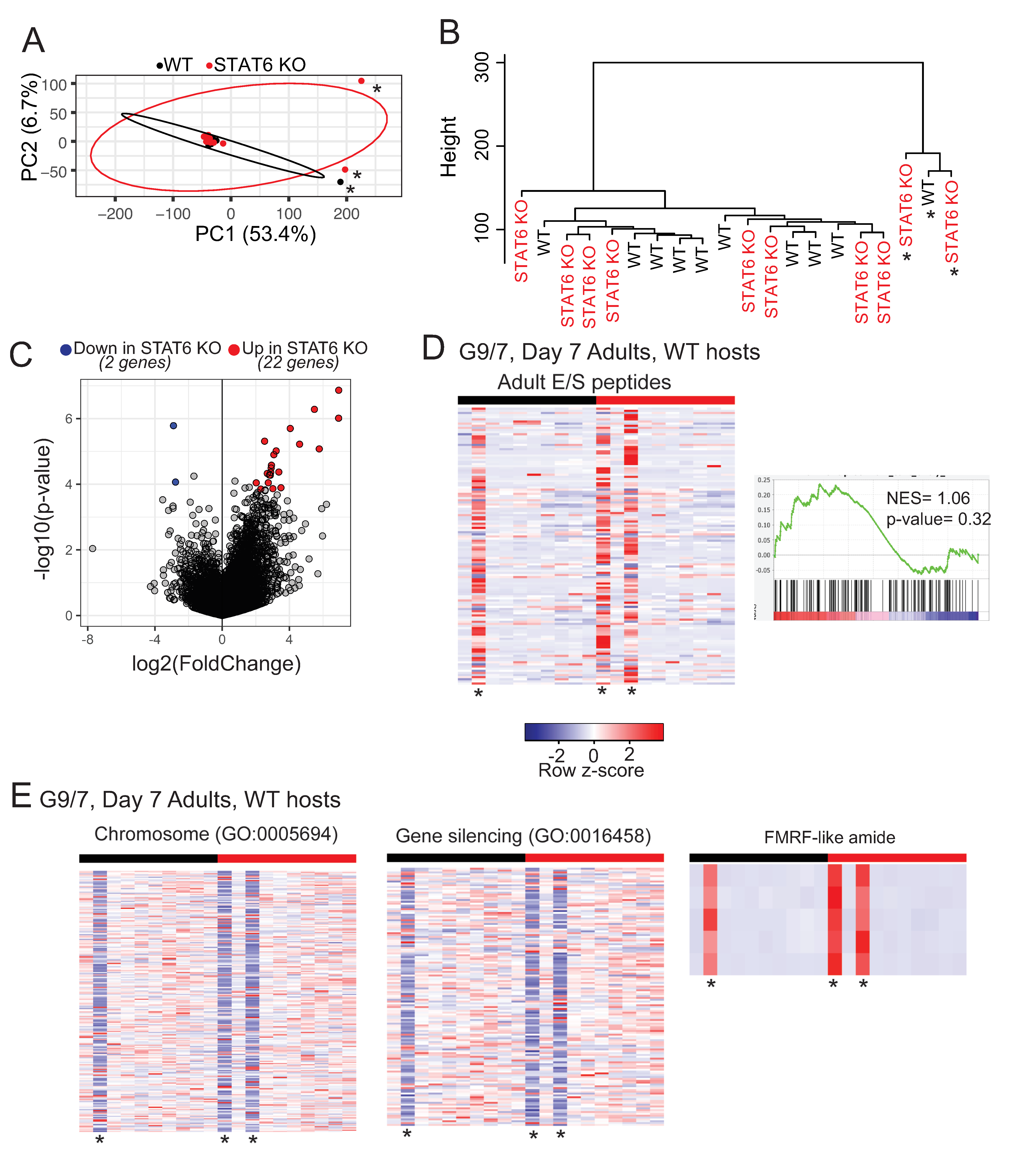

Supplement: S12 Fig — A. PCA plot. B. Hierarchical clustering. C. Volcano plot of differential gene expression results; significance indicated by Log2FC > 2 or < -2, and adjusted p-value < 0.05. D. Heatmap and GSEA enrichment plot for genes encoding adult stage ESP-associated gene categories. E. Heatmaps for expression of genes in Chromosome, Gene Silencing, or FMRFamide-like families. (TIF) [file ppat.1011797.s012.tif]
