# Supplementary material for: Hookworms dynamically respond to loss of Type 2 immune pressure
Source: PLoS Pathog. 2023 Dec 11;19(12):e1011797. doi: 10.1371/journal.ppat.1011797 (PMC10735188; doi:10.1371/journal.ppat.1011797)
Supplement: S1 Table — (DOCX) [file ppat.1011797.s013.docx]

| **KEY RESOURCES TABLE** | | |
| --- | --- | --- |
| REAGENT OR RESOURCE | SOURCE | IDENTIFIER |
| Antibodies | | |
| BUV395^TM^ Rat Anti-Mouse Siglec-F Antibody | BD Biosciences | Cat# 740280. RRID:AB_2740019 |
| BUV563^TM^ Rat Anti-Mouse CD45 Antibody | BD Biosciences | Cat# 752412. RRID:AB_2873124 |
| BUV661^TM^ Rat Anti-Mouse FcϵR Antibody | BD Biosciences | Cat# 751765 RRID: AB_2875742 |
| BUV737 ^TM^ Rat Anti-Mouse CD11b Antibody | BD Biosciences | Cat# 612801. RRID:AB_2738811 |
| e450™ anti-Mouse Ki67Antibody | ThermoFisher | Cat# 48-5698-82 |
| Brilliant Violet 605™ anti-Mouse CD103 Antibody | Biolegend | Cat# 121433 |
| Brilliant Violet 650™ anti-Mouse XCR1 Antibody | Biolegend | Cat# 148220. RRID: AB_2566410 |
| Brilliant Violet 711™ anti-Mouse Ly6G Antibody | Biolegend | Cat# 127643. RRID: AB_2565971 |
| Brilliant Violet 785™ anti-Mouse Ly6C Antibody | Biolegend | Cat# 128041. RRID: AB_2565852 |
| Alexa Fluor® 488 anti-mouse iNOS Antibody | ThermoFisher | Cat# 53-5920-82 |
| PerCP/Cy5.5 anti-Mouse CD86 Antibody | Biolegend | Cat# 105028 |
| APC-conjugated anti-Mouse Arg1 Antibody | R&D systems | Cat# IC3626R |
| Alexa Fluor® 700 anti-mouse I-A/I-E Antibody | Biolegend | Cat# 107622. RRID: AB_493727 |
| APC/Cy7 anti-Mouse CD11c Antibody | Biolegend | Cat# 118218. RRID: AB_2098648 |
| PE conjugated anti-Mouse Relm alpha Antibody | ThermoFisher | Cat# 12-5441-82 |
| PE/Dazzle™594 anti-mouse CD172a (SIRPα) Antibody | Biolegend | Cat# 144016. RRID: AB_2565280 |
| PE/Cyanine5 anti-mouse F4/80 Antibody | Biolegend | Cat# 123112. RRID: AB_893482 |
| PE/Cyanine7 anti-mouse CD64 (FcγRI) Antibody | Biolegend | Cat# 139314. RRID: AB_2563904 |
| BUV395^TM^ Rat Anti-Mouse IL-17 Antibody | BD Biosciences | Cat# 565246. RRID:AB_2722575 |
| Mouse Amphiregulin Biotinylated Antibody | R&D Systems | Cat # BAF989 |
| BUV661 Streptavidin | BD Biosciences | Cat#612979. RRID:AB_2870251 |
| BUV737 ^TM^ Rat Anti-Mouse CD4 Antibody | BD Biosciences | Cat# 612844.RRID:AB_2738811 |
| Brilliant Violet 421™ anti-mouse IL-9 Antibody | Biolegend | Cat# 514109 |
| Brilliant Violet 605™ anti-mouse TCR γ/δ Antibody | Biolegend | Cat# 118129. RRID: AB_2563356 |
| Brilliant Violet 650™ anti-Mouse IFN-γ Antibody | Biolegend | Cat# 505831 |
| Brilliant Violet 711™ anti-Mouse CD90.2 Antibody | Biolegend | Cat# 105349. RRID: AB_2800564 |
| Brilliant Violet 785™ anti-mouse IL-33Rα (IL1RL1, ST2) Antibody | Biolegend | Cat# 145321. RRID: AB_2860702 |
| Gata-3 Monoclonal Antibody (TWAJ), Alexa Fluor™ 488, eBioscience™ | ThermoFisher | Cat# 53-9966-42. RRID: AB_2574493 |
| PerCP/Cy5.5 anti-Mouse CD19 Antibody | Biolegend | Cat# 152406. RRID: AB_2629815 |
| PerCP/Cy5.5 anti-Mouse CD3 Antibody | Biolegend | Cat# 152406. RRID: AB_2629815 |
| PerCP/Cy5.5 anti-Mouse CD11b Antibody | Biolegend | Cat# 152406. RRID: AB_2629815 |
| PerCP/Cy5.5 anti-Mouse CD11c Antibody | Biolegend | Cat# 152406. RRID: AB_2629815 |
| PerCP/Cy5.5 anti-Mouse CD45R Antibody | Biolegend | Cat# 152406. RRID: AB_2629815 |
| PerCP/Cy5.5 anti-Mouse NK1.1 Antibody | Biolegend | Cat# 152406. RRID: AB_2629815 |
| APC anti-mouse IL-5 Antibody | Biolegend | Cat# 505810. RRID: AB_315404 |
| Alexa Fluor® 700 anti-mouse CD44 Antibody | Biolegend | Cat# 103026. |
| Ki-67 Monoclonal Antibody (SolA15), APC-eFluor™ 780, eBioscience™ | ThermoFisher | Cat# 47-5698-82. RRID: AB_2688065 |
| FOXP3 Monoclonal Antibody (FJK-16s), PE, eBioscience™ | ThermoFisher | Cat#12-5773-82.RRID:AB_465936 |
| PE/Dazzle™ 594 anti-mouse CD127 Antibody | Biolegend | Cat# 100762. RRID: AB_22564027 |
| PE/Cyanine5 anti-mouse TCR β chain Antibody | Biolegend | Cat# 109210. RRID: AB_313433 |
| IL-13 Monoclonal Antibody (eBio13A), PE-Cyanine7, eBioscience™ | ThermoFisher | Cat# 25-7133-82. RRID: AB_2573530 |
