# Supplementary material for: Hookworms dynamically respond to loss of Type 2 immune pressure
Source: PLoS Pathog. 2023 Dec 11;19(12):e1011797. doi: 10.1371/journal.ppat.1011797 (PMC10735188; doi:10.1371/journal.ppat.1011797)
Supplement: Attachment — Submitted filename: Ferguson_PLos_Path_Response_to_reviewer_comments 10.24.23.docx [file ppat.1011797.s018.docx]

Reviewer's Responses to Questions

**Part I - Summary**

Please use this section to discuss strengths/weaknesses of study, novelty/significance, general execution and scholarship.

Reviewer #1: This study investigates transcriptional changes in N. brasiliensis when passaged in immunodeficient mice (STAT6KO), to assess how immune pressure affects parasite responses. The authors show that N. brasiliensis can rapidly alter its transcriptional programme in STAT6 KO. These changes do not affect parasite viability in vivo, however they do increase the size of the immune response against the parasite. On infection of STAT6KO-adapted parasites into WT mice, the transcriptional profile of parasites returned to indistinguishable to control parasites.

This studies strengths are around its approach to the experimental system, to see how immune responses affect parasite transcription. Its weaknesses are well addressed in the text, and include bottlenecks of parasite numbers and the effect of immune pressure on proteomic, as well as transcriptional responses of the parasites. However the approach is reasonable and robust, and future studies can address these other issues.

We are grateful to the Reviewer for their review of our manuscript and for their positive comments, and in this revised version have also provided additional data and discussion for future studies to address weaknesses.

Reviewer #2: The manuscript PPATHOGENS-D-23-00743 details that adaptation of N. braziliensis under decreased immunological pressure in STAT6 KO animals. Ferguson et al. elegantly show that Nb maintained in STAT6 KO mice for up to 25 generations shows increased body size and higher egg production. In addition, several transcriptional changes were observed in agreement with lower immunologic pressure on the parasites, including decreased expression of genes involved in motility and excreted and secreted products, that modulate host’s immune system. Increased transcription pathways involved genes associated with reproduction and physiological fitness. Interestingly, these adaptations did not translate to inherited features and the first infection in WT animals, STAT6 KO-adapted parasites returned mostly to their original transcriptional landscape with few exceptions. On the other hand, it seems that adapted parasites lost their ability to downmodulate the immune response, consistently inducing more robust inflammation, which did not translate to fewer viable adult parasites in the host’s gut. This is an sophisticated and well-designed work which brings important information on host-parasite interaction and how immunological pressure can shape a parasite adaptation.

We thank the Reviewer for reviewing our manuscript and their support of our study.

Reviewer #3: This work performed by Ferguson and collaborators investigated whether serial passage of the rodent hookworm Nippostrongylus brasiliensis in STAT6-deficient mice caused changes in parasites over time. By using an improved N. brasiliensis genome, the authors found that these physiological changes observed in the STAT-6 KO-adapted strains corresponded with a dramatic shift in the transcriptional profile, including increased expression of gene pathways associated with parasite persistence. This is interesting story of potential interest to the field, however, there are several issues that need to be addressed to improve the paper, including the fact that the current findings do not sufficient to support the conclusions.

We thank the Reviewer for their thorough feedback, which we have addressed by point-by-point responses below. We believe with this new data and discussion, that this study is stronger and satisfies the Reviewer.

**Part II – Major Issues: Key Experiments Required for Acceptance**

Please use this section to detail the key new experiments or modifications of existing experiments that should be absolutely required to validate study conclusions.

Generally, there should be no more than 3 such required experiments or major modifications for a "Major Revision" recommendation. If more than 3 experiments are necessary to validate the study conclusions, then you are encouraged to recommend "Reject".

Reviewer #1: N/A

Reviewer #2: (No Response)

Reviewer #3: Major comments:

1) The findings presented on this current manuscript don’t show enough evidence to suggest that hookworms alter their transcriptional profile to suit their immediate host immune environment. The most remarkable result is that there is a group of two thousand genes that are downregulated in the adult worms from a parental strain that established the infection in the absence of a type-2 immunity. However, to address the question whether the alteration in those genes were to suit the pressure of the immune response, the authors should have followed up this observation and performed another assay where parental or adapted strains are used in a protective secondary model in WT mice. The analysis of the transcriptional profile of the worms under the pressure of a protective type-2 immune response in comparison with the molecular program of the ones obtained from a secondary model in the STAT-6 KO mice would be very important to associate with those 2,000 genes downregulated in the primary infection.

We have addressed this issue by editing the language of our manuscript to weaken our claim that hookworms alter their transcriptional profile to suit their immediate immune environment. Our data, which provide multiple examples of shifts in transcriptional states correlating more with immediate host immune environment than prior host selection environments, are suggestive that these altered profiles suit the host environments. However, as noted, to strengthen this claim, we would need to test whether similar genes are down-regulated during a secondary infection of STAT6 KO mice vs. WT, which would have enhanced worm-targeting immune responses. While highly interesting, the experiment requested (mRNA-seq of worms from secondary adapted-worm infections of WT vs STAT6 KO mice) is prohibitive in both cost and feasibility to our lab, given the expense of RNA-seq and the challenge of recovering worms from WT hosts given a secondary infection. WT mice with prior exposure to Nb typically do not establish infections in the gut stage because the parasitic larvae are effectively killed in the lung. While we could attempt to boost STAT6-dependent immune response by treating hosts exogenously with recombinant IL-4 cytokine, this would incur further costs and troubleshooting attempts. For these reasons, these experiments are beyond the scope of this current study.

Discussed on lines 469-473, 499-501 of the revised manuscript.

2) As shown in figure 3C, the infection with the parental (rat-adapted) strains into STAT-6 KO mice followed by transcriptional analysis in the G1 parasites revealed the largest differences in their molecular profile when compared with parental strain-infected WT mice. This change was also relevant even when compared with the STAT-6-adapted G14 strain. With that, the authors should demonstrate in Figure 1, what is the EGP, fecundity and parasite burden of a single parental (rat-adapted) strain exposure in STAT-6 KO mice in comparison with the STAT-6 adapted strain?

We have completed a similar experiment once. These data are shown in Reviewer Figure 1, below. We found that EPG from days 5-15 post infection had similar area under the curve (AUC), and that the number of worms in the small intestine at day 15 post infection was the same. We conclude that Parental Nb and STAT6 KO-adapted Nb have similar fitness within STAT6 deficient hosts. Because we failed to see differences in this single experiment, we decided not to investigate whether individual worm fecundity or other features were altered.

Reviewer Figure 1

Reviewer Figure 1: (A) Experimental design. STAT6 KO mice were infected with either parental (WT rat-maintained) or STAT6 KO mouse-adapted (G15) *N. brasiliensis*, and worms or eggs were quantified. (B) Eggs per gram of feces (EPG) for each day post infection. (C) Area under the curve of B. (D) Number of adults in the host small intestine at day 15 post infection. Each circle (C-D) is an individual host; bracketed lines are SEM errors; rectangular bars are the means per condition.

3) In the same line, G1 from STAT-6 KO when infects WT mice also elicit a stronger immune response?

To address this, we performed an experiment measuring the WT mouse immune response to infections with G1 STAT6 KO-adapted Nb relative to WT-G1 controls. We found that there were no differences in the type 2 immune cell proportions in the mesenteric lymph nodes, nor were there differences in type 2 cytokine production by these cells. In addition, we did not find differences in cells producing IFNγ. Indeed, the only difference we observed was a reduction in the proportion of IL17-positive γδT cells in WT mice infected with STAT6 KO G1 worms (Supp. Fig. 9). The myeloid cells similarly did not appear different within the MLN or PECs (Supp. Fig. 9). Collectively, these data suggest that the increased immunogenicity of STAT6 KO-adapted worms emerges only after multiple generations of adaptation.

Discussed on lines 313-324 of the revised manuscript.

4) Is there any difference in the parasite burden of a rat-adapted strain infecting the lungs of WT vs STAT-6 KO mice?

Others have shown that parasite burden in the lung (day 3) is the same between WT and mice lacking STAT6 signaling (IL-4 receptor alpha deficient mice) (1). We have now added data to the manuscript showing no difference in parasite lung parasite burden at 24 hours, 48 hours and 5 days post infection (Supp. Fig. 1).

Discussed on lines 204-206, 398-400 of the revised manuscript.

5) In figure 6 and 7, the characterization of immune response could be more explored by investigating the mechanisms described in the literature associated with the protective immunity against Nippo. Did the authors measure eosinophils? Mucus production?

Yes, we measured eosinophils by gating on live, Siglec F +, CD45+ lymphocytes. We found no difference in the number or proportion of eosinophils in the peritoneal exudate between WT- versus STAT6 KO-adapted infections (Fig. 6, S-T). However, when we examined lung tissue, we found an increased proportion of eosinophils as determined by flow cytometry. This lung tissue also held increased proportions of Arg1+ and RELMa+ macrophages and monocytes, suggesting that these type 2 effector cell types are increased in the lungs. These new data are in Reviewer Figure 2, below.

Reviewer Figure 2

Reviewer Figure 2: (A) Experimental design. WT mice were infected with WT or STAT6 KO host-adapted *N. brasiliensis*, and lungs were analyzed by flow cytometry at day 8 post-infection. (B-J) Cell percentages of parent populations.

Other protective immune mechanisms against hookworms include epithelial remodeling of the small intestine, with increased proliferation of goblet cells and tuft cells. To quantify whether these cell populations have changed, we measured expression of marker genes associated with these cell types from small intestine tissue of WT mice infected with WT or STAT6KO-adapted Nb. We measured expression of MUC2 and MUC5b genes as markers for goblet cells, which would indicate increased mucus production, and of Pou2f3 for tuft cells. For each of these markers, there was no difference in expression (relative to GAPDH) in small intestinal tissue from WT mice infected with WT- versus STAT6 KO-adapted worms (Supp. Fig. 6B-E) . We also measured the length of the small intestine in WT vs STAT6KO Nb-infected mice, and found no significant difference in length, which suggests that the epithelial cell turnover rate between the two groups does not differ by enough to change the length. (Supp. Fig. 6A). While we did not exhaustively test these mechanisms in detail, our MLN and PEC data suggest that the cells within the gut tissue are more type 2 differentiated in the STAT6 KO Nb infection condition with respect to cytokine production and macrophage polarization; however, other intestinal epithelial cell effector mechanisms are not impacted. M2 macrophages, neutrophils and eosinophils are all understood to limit parasite burden in the lungs and gut; this is likely to be due to trapping, killing, and nutrient deprivation, particularly against the larval stage (2,3). However, recent work by Westermann et al. (4) demonstrates that STAT6-dependent functions of AA macrophages are dispensable against enteric helminths, while STAT6-dependent IEC functions are necessary for limiting parasite burden. Our data fit with this understanding: we found that STAT6 KO-adapted worms did not have reduced fitness within WT hosts, despite initiating a stronger cytokine and AAM response. Our data furthermore suggest that the IEC responses are similar (by tuft cell and goblet cell markers, and SI length) in these conditions.

Discussed on lines 299-304 and 435-437-494 of the revised manuscript.

**Part III – Minor Issues: Editorial and Data Presentation Modifications**
Please use this section for editorial suggestions as well as relatively minor modifications of existing data that would enhance clarity.

Reviewer #1:

Figure 1D: WT vs STAT6 KO data is in the opposite order to other figures

Figure 1D was re-ordered to match other figures.

Figure 2A: STAT6KO worm image contains a section of background medium (outside of the boundary of the worm edge), making it look redder and bigger, and is slightly misleading.

This may be a misunderstanding by the reviewer. In this figure, we have rectangular-cropped images, whose background is clear agarose that appears almost entirely white. The bright red regions that appear along the perimeter of the worm are indeed part of the worm body. To emphasize this point, we have updated the methods and added arrows to the images marking the edge of the worm.

Lines 234-267: The genes mentioned here could be highlighted in volcano plots or shown as raw data. As a resource for the community, this raw data should be made available.

We have added supplementary files (Supp. Files 3-5), containing raw differential gene expression data (including log2FC, p-value, adjusted p-value, and normalized expression values), for all genes analyzed, and select genes of interest (including gene sets: FMRFamide-related peptide-like [IPR002544], Neuropeptide signaling (GO:0007218), cytochrome p450, HSP20-like chaperone, ESP associated genes)

Supp Fig 7A-C: FcERI+ cells could be mast cells or DCs, could presumably tell by looking at SSC to see whether there are the same populations, or increased recruitment of a specific cell type?

We were able to subset FcER1+ cells based on Class II and CD11c expression. MHC class II CD11c double positive were defined as dendritic cells, MHC II positive CD11c negative were defined as B cells, and double negative cells that were further gated on large size and granularity (FSC, SSC) were mast cells. Shown in Reviewer Figure 3, below, this new gating resulted in most FcER1+ cells being classified as mast cells, followed by B cells and Dendritic cells. Cell numbers for each of these populations was increased in the STAT6 KO-adapted condition.

Reviewer Figure 3

Reviewer Figure 3: A. Gating for identification of FcER subsets. B. Pie charts showing proportions of FcER+ cell subsets. C-F. Cell numbers of subsets. Each circle is data from a one mouse, bars are group means, error bars are SEM. * p-value < 0.05, *** < 0.005.

There is a placeholder statement for the data availability, presumably this will be corrected later.

We have indeed corrected this. Our revised manuscript now gives a full set of GenBank accession numbers for the overall genomic data, the improved genome assembly with its protein-coding gene set, and the RNA-seq data used in this study. All of these links are live and public as of October 2023. The exact genomic sequences (proteins, coding DNAs, genomic sequences, and GFF annotations) used in our analysis prior to GenBank submission have been permanently archived at OSF.io, with a live web address for this OSF.io archive also given.

Reviewer #2: The authors found that STAT6 KO-conditioned parasites induce higher inflammation when used to infect WT animals, however, discuss little about the mechanisms. They can discuss parasite-related factors, since they have the transcriptomics and know the few genes that are still differentially expressed.

Our data suggests that STAT6 KO-adapted worms have some quality that renders them more immunogenic than WT-adapted worms. It is likely that one or more of excreted-secreted (ES) products (proteins or other molecules) is responsible for this change, given that hookworm ES products can either promote inflammation or dampen it. Hence, it is possible that our observed difference is due to either increased levels of an inflammatory ES product and/or decreased levels of an immune-dampening product. In our data, we found that few genes are differentially expressed at the adult stage. However, the strongest difference was STAT6 KO-decreased expression of genes whose products are predicted to be secreted proteases of the Astacin family. These genes could serve as candidates for study as having immune-dampening properties. The immunogenic property of STAT6 KO-adapted worms is also likely present in earlier life cycle stages, like L4 worms in the lung. We did not directly analyze the transcriptome or fitness of this stage of the infection for this project. However, in one experiment, we observed an altered immune cell profile of the lungs, which were harvested at day 8 post infection in STAT6 KO-adapted worm infections. The lung tissue had increases in type 2 lymphocytes like IL-5-producing Th2 cells (Reviewer Figure 4).

Reviewer Figure 4

Reviewer Figure 4: (A) Experimental design. (B) Percent of parent IL-5 positive Th2 cells. (C) Number of IL-5 positive Th2 cells. Circles are measurements from individual mice; solid bars are means; error lines are SEM. 1 experimental repeat. **p-value < 0.005

In addition, this tissue had increased eosinophils, and arginase-expressing macrophages (Reviewer Figure 2). This suggests that the lung stage of infection was also more immunogenic. Further work will address whether the transcriptional profile of the STAT6 KO-adapted L4 lung stage worms is altered during WT infection and potentially added to this increased immunogenicity.

Discussed on lines 354-358 and 447-451, 479-482 of the revised manuscript.

In addition, they could provide some information about how these parasites regulate anti-inflammatory mechanisms on the host. They have FOXP3 on the flow cytometry panels, but do not show any information on Tregs, which are relevant to these findings.

We explored whether the Treg population was different in WT mice infected with STAT6 KO-adapted Nb versus WT controls. Unfortunately, over the three independent experiments performed, the results were inconsistent. In one experiment, mesenteric lymph node Tregs were decreased in the STAT6KO condition; but when we repeated this experiment, we observed the opposite trend and no difference in a third repeat. These data are provided in Reviewer Figure 4, below.

Reviewer Figure 4

Reviewer Figure 4: (A) Experimental design. WT mice were infected with WT- or STAT6 KO-adapted *N. brasiliensis*, and host mesenteric lymph nodes were analyzed by flow cytometry at day 8 post infection. (B, D, F) Percentages of Tregs. (C, E, G) Number of Tregs.

Based on this, we cannot conclude that the STAT6 KO-adapted worms induce consistent differences in Tregs; even though there were differences in inflammatory cytokines for each of these experiments. Hookworm ES proteins can promote immune dampening by directly promoting proliferation of Tregs (5). They are also proposed to dampen host inflammation by directly inhibiting neutrophils (6) and macrophages (7). Adult hookworms are thought to express C-type lectins in order to dampen inflammation during feeding by antagonizing host lectins (8). In addition, ES can deliver other molecules like miRNA via extracellular vesicles, to potentially control the expression of host immune genes (9). Given the increase in type 2 cytokine levels, and alternatively activated macrophages that we observed, the key differences in our STAT6 KO-adapted worms may be in their ability to suppress the sensing and/or amplification of type 2 immunity, or perhaps, the differentiation of M2 macrophages. Interestingly, we found that some of the down-regulated ES encoding genes in STAT6 KO-adapted worms derived from STAT6 KO hosts corresponded to molecules that had been previously characterized as immunomodulatory. However, the differentially-expressed genes from STAT6 KO-adapted worms within WT hosts did not correspond to known or suspected immune-dampening functions. Indeed, prior research on Astacin-like metallopeptidases suggests an immunogenic function (10). Our data therefore suggests that one or more yet-to-be-characterized ES products may be responsible.

Discussed on lines 447-451 479-482 of the revised manuscript.

Another intriguing finding is that STAT6KO-adapted L4 showed minimal adaptation (G1), when compared to adult worms at the same passage. This is coherent with the finding that despite such adaptation in adults, no difference in survival is observed when adapted worms are reintroduced to WT mice. It would be interesting if the authors could provide some discussion on the different mechanisms at play in the lungs and intestines that are dependent on STAT 6 and why there is such a big difference between L4 and adults, or would it be just because L4 activated less genes during their development and are less likely to modulate large numbers of genes based on host’s immune pressure.

Our findings suggest that STAT6-dependent immune pressure does not impact worm larvae in the lung to the same extent as it does adult worms in the gut. The key STAT6-driven immune responses that are purported to impact worms include alternatively activated macrophages, which bind to larval stages of worms to trap them in the tissue. Because worms are in the lungs for a relatively short time, it is possible that this developmental stage of *N. brasiliensis* does not have enough time to be affected by the immune pressure and alter their transcriptome accordingly. On the other hand, the STAT6-dependent mechanisms for controlling worms in the small intestine include increased epithelial cell turnover, increased tuft cell expansion leading to type 2 immunity, increased mucus production, and increased peristalsis; all of these mechanisms increase in strength over the course of infection, and function to eject the worms from the GI lumen by day 9 post infection. We find that worms are not dramatically altered in their transcriptional profile in the gut at day 5 post infection, but do have an altered profile at day 7 post infection; this is consistent with a longer exposure to gut STAT6 dependent mechanisms as a likely key driver hookworm transcriptional differences. Thus, our data suggests that the difference in lung versus gut stage worm transcriptional profile is due to both the length of time the worm is exposed to these tissue environments and the strength of immune pressure to which the worms are subjected.

Discussed lines 393-409

A minor comment is that it is hard to distinguish blue dots from black dots in Figure 3 and alike. Perhaps using a lighter blue would facilitate to appreciate the results easily.

Volcano plots have been updated with a lighter shade of blue.

Reviewer #3: (No Response)

PLOS authors have the option to publish the peer review history of their article ([what does this mean?](https://journals.plos.org/plospathogens/s/editorial-and-peer-review-process#loc-peer-review-history)). If published, this will include your full peer review and any attached files.


If you choose “no”, your identity will remain anonymous but your review may still be made public.


**Do you want your identity to be public for this peer review?** For information about this choice, including consent withdrawal, please see our [Privacy Policy](https://www.plos.org/privacy-policy).

Reviewer #1: No

Reviewer #2: **Yes:** Helton Santiago

Reviewer #3: No

Figure Files:

While revising your submission, please upload your figure files to the Preflight Analysis and Conversion Engine (PACE) digital diagnostic tool, [https://pacev2.apexcovantage.com](https://pacev2.apexcovantage.com/). PACE helps ensure that figures meet PLOS requirements. To use PACE, you must first register as a user. Then, login and navigate to the UPLOAD tab, where you will find detailed instructions on how to use the tool. If you encounter any issues or have any questions when using PACE, please email us at [figures@plos.org](mailto:figures@plos.org).

Data Requirements:

Please note that, as a condition of publication, PLOS' data policy requires that you make available all data used to draw the conclusions outlined in your manuscript. Data must be deposited in an appropriate repository, included within the body of the manuscript, or uploaded as supporting information. This includes all numerical values that were used to generate graphs, histograms etc.. For an example see here on PLOS Biology: http://www.plosbiology.org/article/info%3Adoi%2F10.1371%2Fjournal.pbio.1001908#s5.

Reproducibility:

To enhance the reproducibility of your results, we recommend that you deposit your laboratory protocols in protocols.io, where a protocol can be assigned its own identifier (DOI) such that it can be cited independently in the future. Additionally, PLOS ONE offers an option to publish peer-reviewed clinical study protocols. Read more information on sharing protocols at <https://plos.org/protocols?utm_medium=editorial-email&utm_source=authorletters&utm_campaign=protocols>

References:

1.	Horsnell WGC, Vira A, Kirstein F, Mearns H, Hoving JC, Cutler AJ, et al. IL-4Rα-responsive smooth muscle cells contribute to initiation of TH2 immunity and pulmonary pathology in Nippostrongylus brasiliensis infections. Mucosal Immunol. 2011 Jan;4(1):83–92.

2.	Chen F, Wu W, Millman A, Craft JF, Chen E, Patel N, et al. Neutrophils prime a long-lived effector macrophage phenotype that mediates accelerated helminth expulsion. Nat Immunol. 2014 Oct;15(10):938–46.

3.	Bouchery T, Kyle R, Camberis M, Shepherd A, Filbey K, Smith A, et al. ILC2s and T cells cooperate to ensure maintenance of M2 macrophages for lung immunity against hookworms. Nat Commun. 2015 Apr 27;6(1):6970.

4.	Westermann S, Schubart C, Dietschmann A, Castiglione K, Radtke D, Voehringer D. Th2-dependent STAT6-regulated genes in intestinal epithelial cells mediate larval trapping during secondary Heligmosomoides polygyrus bakeri infection. PLoS Pathog. 2023 Apr;19(4):e1011296.

5.	Navarro S, Pickering DA, Ferreira IB, Jones L, Ryan S, Troy S, et al. Hookworm recombinant protein promotes regulatory T cell responses that suppress experimental asthma. Science Translational Medicine. 2016 Oct 26;8(362):362ra143-362ra143.

6.	Moyle M, Foster DL, McGrath DE, Brown SM, Laroche Y, De Meutter J, et al. A hookworm glycoprotein that inhibits neutrophil function is a ligand of the integrin CD11b/CD18. J Biol Chem. 1994 Apr 1;269(13):10008–15.

7.	Cho Y, Jones BF, Vermeire JJ, Leng L, DiFedele L, Harrison LM, et al. Structural and functional characterization of a secreted hookworm Macrophage Migration Inhibitory Factor (MIF) that interacts with the human MIF receptor CD74. J Biol Chem. 2007 Aug 10;282(32):23447–56.

8.	Loukas A, Prociv P. Immune responses in hookworm infections. Clin Microbiol Rev. 2001 Oct;14(4):689–703, table of contents.

9.	Eichenberger RM, Ryan S, Jones L, Buitrago G, Polster R, Montes de Oca M, et al. Hookworm Secreted Extracellular Vesicles Interact With Host Cells and Prevent Inducible Colitis in Mice. Front Immunol. 2018;9:850.

10.	Bąska P, Wiśniewski M, Krzyżowska M, Długosz E, Zygner W, Górski P, et al. Molecular cloning and characterisation of in vitro immune response against astacin-like metalloprotease Ace-MTP-2 from Ancylostoma ceylanicum. Experimental Parasitology. 2013 Apr 1;133(4):472–82.
